# An Evaluation of Electromagnetic Exposure While Using Ultra-High Frequency Radiofrequency Identification (UHF RFID) Guns

**DOI:** 10.3390/s20010202

**Published:** 2019-12-30

**Authors:** Patryk Zradziński, Jolanta Karpowicz, Krzysztof Gryz, Victoria Ramos

**Affiliations:** 1 Laboratory of Electromagnetic Hazards, Central Institute for Labour Protection—National Research Institute (CIOP-PIB), ul. Czerniakowska 16, 00-701 Warszawa, Poland; jokar@ciop.pl (J.K.); krgry@ciop.pl (K.G.); 2 Telemedicine and e-Health Research Unit, Instituto de Salud Carlos III, 28025 Madrid, Spain; vramos@isciii.es

**Keywords:** biomedical engineering, environmental engineering, numerical simulations, radiofrequency sensor, occupational exposure, public health, specific energy absorption rate (SAR)

## Abstract

The aim is to evaluate specific absorption rate (SAR) values from exposure near handheld ultra-high frequency radiofrequency identification readers (UHF RFID guns—small electronic devices, or even portable computers with relevant accessories—emitting up to several watts of electromagnetic field (EMF) to search for RFID sensors (tags) attached to marked objects), in order to test the hypothesis that they have an insignificant environmental influence. Simulations of SAR in adult male and female models in seven exposure scenarios (gun near the head, arm, chest, hip/thigh of the operator searching for tags, or near to the chest and arm of the scanned person or a bystander). The results showed EMF exposure compliant with SAR limits for general public exposure (ICNIRP/European Recommendation 1999/519/EC) at emissions up to 1 W (reading range 3.5–11 m, depending on tag sensitivity). In the worst-case scenario, guns with a reading range exceeding 5 m (>2 W emission) may cause an SAR exceeding the general public limits in the palm of the user and the torso of the user, a bystander, or a scanned person; occupational exposure limits may be exceeded when emission >5 W. Users of electronic medical implants and pregnant women should be treated as individuals at particular risk in close proximity to guns, even at emissions of 1 W. Only UHF RFID guns emitting below 1 W may be considered as environmentally insignificant EMF sources.

## 1. Introduction

### 1.1. RFID Technology

Radio frequency identification (RFID) is the most common and fastest-growing wireless technology of automatic identification and data capture (AIDC) that identifies and tracks RFID sensors (tags) attached to objects. The tags contain electronically stored information that includes a code defined for each tag individually (predefined before the use of the tag, or modified over the life of the tagged object) [1]. Two-way radio transmitter-receivers, called interrogators or readers, send electromagnetic field (EMF) to the tag and read its response, with the possibility of modifying the data stored in the tag. An RFID reader is responsible for sensing (reading) and writing data on tags and for powering up the most popular passive tags, which collect energy by wireless interaction from EMF emitted by a nearby RFID reader [1]. Unlike a barcode, the tag does not need to be within the line of sight of the reader, so it may be embedded inside the tracked object.

The advantages of this technology mean that it has many and rapidly developing applications in shops, warehouses, libraries, enterprises, and even medical centers, prisons, and the public environment, such as:

- managing contactless cards (e.g., tolls, access control to rooms or buildings, public transport tickets, payment),
- controlling time,
- managing, controlling, or monitoring objects (marking goods, wares, devices, books, document tracking in offices, or even patients, biological materials, pharmaceuticals, prisoners or animals).

A physical RFID tag may be incorporated with browser-based software to increase its efficacy as a part of a real-time locating system (known as RTLS). This software allows for different groups or specific personnel to see, store, and archive real-time data relevant to each piece of tracked equipment or personnel, to make use of historical reporting functionality, and to demonstrate compliance with various industry regulations. RTLS provides a powerful data collection tool for facilities seeking to improve operational efficiency and reduce costs of various processes.

An RFID system may replace or supplement barcode systems and may offer another faster method of inventory management and self-service checkout by patrons (e.g., in a public environment such as shops and libraries; over 30 million library items worldwide now contain RFID tags, including some in the Vatican Library in Rome [2]). It can also act as a security device, taking the place of the more traditional electromagnetic security strips [3]. 

Quite a new but very effective, rapidly growing example is the use of RFID technology in healthcare to increase visibility and efficiency and to gather data around relevant interactions. RFID tracking solutions are able to help healthcare facilities manage pharmaceuticals and mobile medical equipment (large or small equipment, e.g., by improving the control of its location, managing single-use items or surgical tools that need sterilization and inventory after the surgical intervention, and even laundry tracking); improve patient and attendee workflow by verifying information on their status or location; monitor environmental conditions and protect patients, staff, and visitors from infection or other hazards; and access control. 

Hospitals have been among the first users to combine both active (with a battery which allows a reach of longer distance and emits stronger EMF) and passive (without battery) RFID tags. Many successful deployments in the healthcare industry have been cited where active RFID technology is used to track high-value or frequently moved items, and passive RFID technology is used to track smaller, lower-cost items that only need room-level (short distance) identification [4]. For example, medical facility rooms can collect data from transmissions of RFID tags worn by patients and employees, e.g., inside the identification badges, as well as from tags assigned to mobile medical devices [5].

The most commonly used RFID systems in the world are LF (low frequency band: 30–300 kHz; operating typically at 125 kHz), HF (high frequency band: 3–30 MHz; operating typically at 13.56 MHz frequency), UHF (ultra-high frequency band: 300–1000 MHz; operating in the frequency range 860–965 MHz (in Europe, 865–868 MHz) for passive tags and 443 MHz for active tags), and SHF (super-high frequency bands: 2.400–2.4835 GHz and 5.725–5.875 GHz; operating typically at 2.45 and 5.8 MHz frequency) [1]. They may use fixed readers equipped in antennas of various dimensions, from several centimeters up to one meter. Alternatively, handheld readers usually operate with antennas of dimensions smaller than 20 cm, which are built into small portable electronic intelligent devices or are a kind of periphery accessory attached to small portable computers, tablets, or palmtops.

### 1.2. Realistic Scenarios of Exposure to EMF Near RFID Devices

The large number of possible applications of RFID technology has resulted in the operators of such devices, along with other people present in the vicinity, being widely exposed to EMF, especially anyone scanned by a reader to check whether any tag is present on the body or clothing. Typically, the operation of such technology is related to the use of RFID devices close to the body. Many applications require the use of handheld RFID readers, recognized as RFID guns. Special attention to EMF exposure is needed when RFID technology is used in the environment accessible to the general public, where an evaluation of the exposure of workers (users of technology) and bystanders (e.g., customers or passengers) may be required.

**Figure 1:**
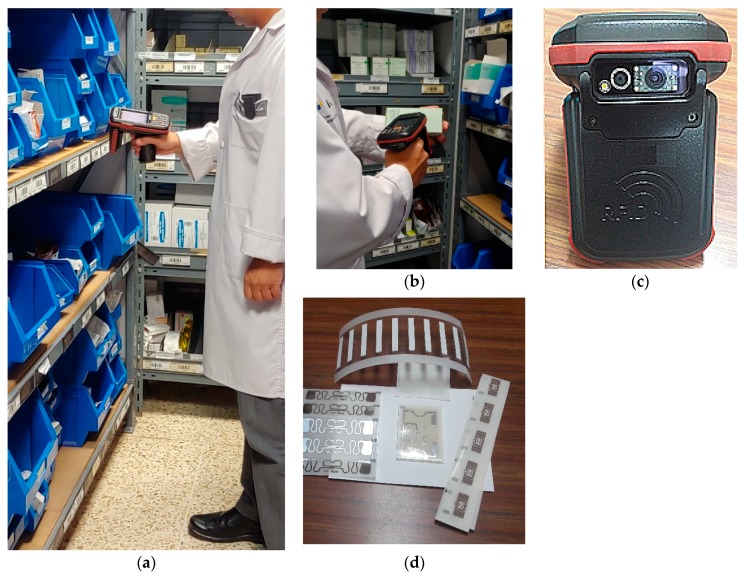
Examples of a handheld UHF RFID gun reader and tags: (**a,b**) the position of the operator scanning wares marked by RFID tags, (**c**) RFID gun, (**d**) RFID tags.

The RFID gun is normally kept in hand or is suspended on the shoulder using special suspenders. While it is used, it is held in the hands at the height of the hips up to the chest (Figure 1). The result is that EMF emitted by the active gun antenna affects the operator’s palm, chest, head, and hip/legs. When the RFID gun is used to scan any person, or is unintentionally activated near bystanders in the vicinity, they may receive localized EMF exposure near the gun’s position, most probably at chest height. As is shown in Figure 1a,b, the reader’s device will have a position close to the user’s thorax, with the separation allowed by the forearm (e.g., 15–20 cm). In the case of readings made with the arm extended, the separation of the body would be greater and the operator would receive less EMF exposure, but the performance of the work in this way causes a higher musculoskeletal load (the weight of the RFID gun may be at the level of 0.7 kg) [6,7].

### 1.3. EMF Exposure Metrics and Evaluation 

When discussing the protection of humans against electromagnetic hazards, the general public is defined as individuals of all ages and of different health statuses, which may include particularly vulnerable individuals who may have no knowledge of or control over their exposure to EMF. Whereas in the analysis of workers’ EMF exposure, it is usually hypothesized that exposure in the work environment touches only healthy adults who are informed about exposure conditions and trained in how to avoid EMF hazards. These differences may be interpreted towards the need to include more stringent restrictions for the general public as members of the general public would not be suitably trained to mitigate harm from EMF exposure or may not have the capacity to do so.

The international guidelines provided by the ICNIRP (the International Commission on Non-ionizing Radiation Protection), widely used over two decades worldwide, deem the thermal effects from radiofrequency EMF exposure to be the main potentially harmful exposure effect that needs to be limited in order to ensure compliance with the minimum safety requirements on workers and the general public [8]. 

Within the 100 kHz to 6 GHz frequency range, the metrics of thermal effects used by ICNIRP is recognized as SAR values (specific energy absorption rate, expressed in watts of absorbed EMF energy per kilogram of exposed tissue, W/kg) and split into two regions: “Head and Torso”—the head, eye, abdomen, back, thorax and pelvis, and “Limbs”—the upper arm, forearm, hand, thigh, leg and foot. The SAR is evaluated as averaged over the entire exposed body (recognised as whole-body averaged SAR—WBSAR) and also as localized SAR averaged in particular smaller parts of the exposed body (over 10 g mass of any continuous tissue—SAR10g) [9]. SAR values are evaluated by the numerical modeling of EMF interaction between the model of the EMF source and the heterogeneous anatomical body model, usually with the use of finite-difference time-domain (FDTD) numerical code compliant with the requirements of the relevant international standard.

The limit values of SAR are recognized to be basic restrictions in the system of limits defining criteria for an evaluation of EMF exposure parameters. Because of the limited availability of SAR values characterizing EMF exposure in the real environment, the secondary metrics characterizing EMF exposure were derived from the relationship between the level of quasi-homogeneous EMF exposure and SAR values—the strength of the electric and magnetic fields (E, expressed in volt per meter, V/m, and H, expressed in ampere per meter, A/m) affecting the human body—recognized as reference levels. When the EMF exposure is localized, i.e., the level of exposure averaged over the body significantly differs from the maximum exposure of any part of the body, the direct use of an SAR evaluation is recommended by ICNIRP [8]. There is no general criterion on how to recognize localized exposure needing evaluation with respect to basic restrictions, but a distance between the EMF source and the exposed body shorter than 20 cm is mentioned in several papers [10,11]. In the case of localized EMF exposure, the exposure effects in the body (SAR values) may be compliant with the basic restrictions limits even at EMF exposures significantly exceeding the reference levels.

The basic restriction (WBSAR of 0.4 W/kg) for occupational exposure was established taking into account scientific uncertainty as well as differences in thermal baselines, the thermoregulation ability and body core temperature across the population, and a reduction factor of 10 of the threshold of exposure level corresponding to an increase in body core temperature of 1 °C (WBSAR of 4 W/kg averaged over the entire body mass and a 6-min interval). However, the basic restriction for the general public was established with the use of a reduction factor of 50 (WBSAR of 0.08 W/kg).

The limits provided by the ICNIRP for basic restrictions and reference levels applicable for an evaluation of EMF exposure at a frequency band used by UHF RFID systems (at center frequency 900 MHz in that band) are summarised in Table 1. EMF exposure limitations published by ICNIRP were used in various legislative documents dealing with the formal requirements regarding protection against electromagnetic hazards; for example in the non-binding Council Recommendation 1999/519/EC and binding Directive 2013/35/EU, currently in force in Europe; however, lower exposure limits were set in various countries (for example, in China and India) [8,12,13].

### 1.4. EMF Emissions from RFID Devices

EMF emissions from an RFID gun (its activation) are at a maximum level of radiated power and continuous in time only during the test mode, which may last up to several minutes and is fully controlled by the test software. In real use mode, the gun is activated only as long as the activation button is pressed, which would seldom last for six consecutive minutes, although it can be much longer than 6 min in discontinuous use.

In accordance with ETSI/EN 302-208 V3.1.1:2016, being a harmonized standard with Directive 2014/53/EU (recognized as the RED directive) in the case of UHF RFID systems, the limit of effective radiated power (ERP) from the antenna is 2 W (for frequency band 865–868 MHz) or 4 W (for frequency band 915–928 MHz) for devices where use does not require special permission (devices equipped with more powerful antennas may be used only when adequate administrative permission has been obtained) [14,15]. There is not much investigative data reported in the literature regarding EMF exposure and the evaluation of the biophysical effects of exposure to RFID systems. Reports in the literature on RFID technology are mainly concerned with the design of low cost, technologically simplified antennas with circular polarisation, allowing the reading of tags from the greatest possible range. Fiocchi et al. [16] calculated the SARs (6-min averaged) for various anatomical human body models exposed to EMF emitted by a UHF RFID reader with an antenna of circular polarisation in various locations and distances (10, 20, 50 cm) against human body models. They concluded that the highest localised SAR10g found in pregnant females was close to the ICNIRP general public limit for 1 W antenna radiated power. In the perspective of the mentioned results of the studies, at the maximum permissible emission from an unregistered UHF RFID system (2 W radiated power in Europe, 4 W in, e.g., North America), the SAR values may be exceeded when exposure lasts for several minutes, at least with respect to general public exposure limits. Near the more powerful readers, SAR values may be exceeded in an exposure time shorter than the 6 min considered above.

### 1.5. Aim

The aim of this study is to evaluate the SAR values in the body of a person present near handheld UHF RFID readers (recognized as UHF RFID guns) in order to test the hypothesis that the most popular UHF guns may be considered as EMF sources without a significant environmental influence, evaluated with respect to the EMF influence on humans (the operators of RFID guns, or the scanned persons or bystanders) and relevant international safety guidelines in various exposure scenarios mimicking the most typical exposure situations.

## 2. Materials and Methods

Many applications require the use of the handheld RFID guns investigated in this study (in many applications based on portable computers equipped with the relevant software, accessories and external antenna), and thus, there is exposure to strong heterogeneous EMF. Such cases require an assessment of the biophysical effects of the exposure (characterized by SAR values). SAR values are accessible by laboratory measurements in simplified exposure scenarios through the use of homogeneous body phantoms, but for the evaluation of more realistic exposure scenarios, numerical calculations using anthropo-morphing heterogeneous body phantoms are widely applied [17,18,19]. SAR values are evaluated with respect to the ICNIRP limits set for 900 MHz (approximately the center frequency from the UHF RFID band), as shown in Table 1. The reasonably foreseeable conditions of exposure should be based on realistic exposure and/or installation parameters representative of all readily-predictable human and system behavior, such as the duration of exposure, the time-varying of transmitted power, operated frequency bands, and time averaging, as defined in safety guidelines [12]. All the intended operating conditions, as well as the reasonably foreseeable conditions of human exposure from the equipment, should be taken into account in the exposure evaluation required by provisions of Directive 2014/53/EU [14].

### 2.1. Numerical Models of EMF Sources

**Figure 2:**
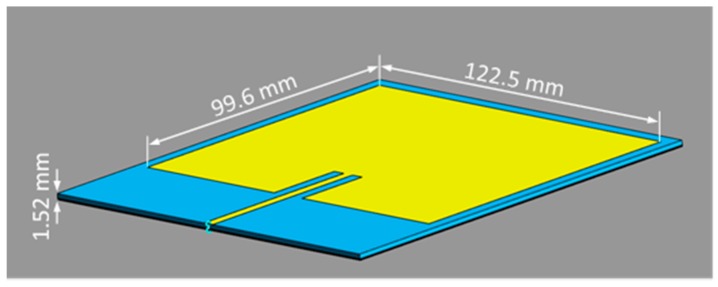
Numerical model of the UHF RFID reader used in investigations: microstrip patch antenna operating at 865 MHz (copper patch of 99.6 × 122.5 × 0.035 mm placed on Rogers R3003 substrate of 131.5 × 138.3 × 1.52 mm, PEC ground thickness of 0.035 mm).

Investigations cover the model of a UHF RFID gun with a microstrip patch antenna designed to operate at 865 MHz (a patch of 99.6 × 122.5 mm placed on substrate and ground of 131.5 × 138.3 mm) (Figure 2). Copper with a thickness of 0.035 mm was used for the patch; Rogers RO3003 with a thickness of 1.52 mm was used as substrate; ground with a thickness of 0.035 mm was modelled as PEC (perfect electric conductor). Elements related to the antenna housing, its fixation, gun handle, and monitor were not included in the numerical model.

The parameters of typically used UHF RFID guns are radiated power up to 1 W, a reading range up to 7 m (readers with a longer reading range are also used—they emit a higher power, up to several watts).

### 2.2. Exposure Scenarios

The investigated exposure scenarios used in this study considered that the antenna was located vertically (similar to the position of the external antenna in the RFID gun shown in Figure 1).

**Figure 3:**
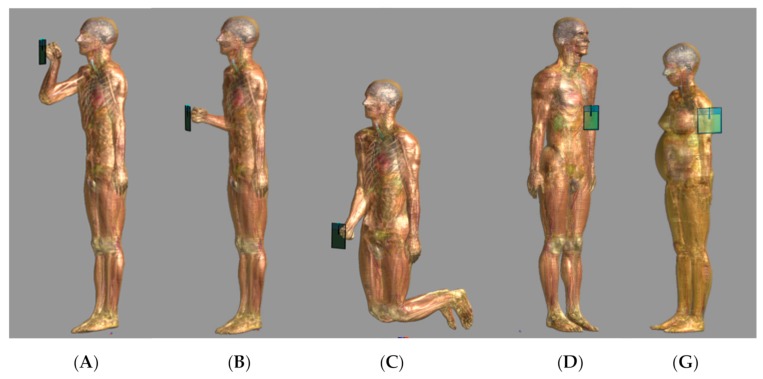
Exposure scenarios: (1) exposure of the operator holding an RFID gun in the hand (**A**—in front of the face, 20 cm away from it; **B**—in front of the chest, 20 cm away from it; **C**—holding the arm lowered, 16 cm away from the abdomen), and (2) exposure of people approaching the RFID gun held by the operator in scenario B (**D**—in front of the chest (male model), 5 cm away; corresponding scenario involving the pregnant female model is F (not shown in the figure); **G**—at the side of the body, at the height of the chest (pregnant female model), at a distance of 5 cm to the arm; corresponding scenario involving the male model is E (not shown in the figure)).

The exposure scenarios include (Figure 3):

(1) Exposure of the operator of the RFID gun (3 exposure scenarios analyzed with the use of a male body model) to be evaluated with respect to the exposure limits set for worker exposure when the worker is holding a gun in hand or with respect to exposure limits set for general public exposure when a gun is used by a person without a regular employment contract, or without the mentioned understanding of electromagnetic hazards:
  - Scenario A—in front of the face, 20 cm away from it, reading tags located at the height of 160–200 cm (A-Head-20cm);
  - Scenario B—in front of the chest, 20 cm away from it, reading tags located at the height of 100–140 cm (B-Chest-20cm);
  - Scenario C—holding the arm lowered, 16 cm away from abdomen, the user kneeling, reading tags located at the height of 30–70 cm (C-Hip-16cm).
(2) Exposure of people approaching the operator of an RFID gun as scanned person or bystander (4 exposure scenarios analyzed with the use of a male body model and a pregnant female model) to be evaluated with respect to the exposure limits set for general public exposure or worker exposure (depending on the purpose of the use of the RFID gun), when a gun is located:
  - Scenario D—in front of the chest of the scanned person or bystander (male model), at the same height as in scenario B, 5 cm away (D-Chest-5cm);
  - Scenario E—at a side of the body of the scanned person or bystander (male model), at the same height as in scenario B, at a distance of 5 cm to the arm (E-Side-5cm); not shown in Figure 3, comparable to scenario G analyzed with the female model;
  - Scenario F—in front of the chest of the scanned person or bystander (pregnant female model), at the same height as in scenario B, 5 cm away (F-Chest-5cm); not shown in Figure 3, comparable to scenario D analyzed with the male model;
  - Scenario G—at a side of the body of the scanned person or bystander (pregnant female model), at the same height as in scenario B, at a distance of 5 cm to the arm (G-Side-5cm).

Exposure scenarios D, E, F, and G represent the exposure of a person scanned by the operator who uses a RFID gun in exposure scenario position B.

### 2.3. Human Body Numerical Model

An anatomical numerical male model (Duke) and Pregnant Woman II (a female in the 7^th^ month of pregnancy), composed of over 300 tissues/organs (such as skin, fat, bones, muscles) developed by the IT’IS Foundation (The Foundation for Research on Information Technologies in Society (IT’IS), Switzerland), were used in the investigations. The male model has a height of 177 cm, a weight of 70.2 kg, and a body mass index (BMI) of 22.4. Its parameters are very close to ICRP 110 Adult Reference Computational Phantoms, which for male models have a height of 176 cm and a weight of 73 kg [17]. The Duke model allows the body posture to be changed at the main joints of the body, e.g., knee, elbow, hip, shoulders, fingers. The Pregnant Woman II model has a height of 163 cm, a weight of 63.2 kg, and a BMI of 23.8. The body posture of Pregnant Woman II cannot be changed.

### 2.4. Numerical Simulations

Numerical simulations were carried out by Sim4Life software (Zurich Med Tech, Switzerland) using finite difference time-domain solvers (FDTD). The FDTD method is the solution of Maxwell’s curl equation in the time domain [20]. The finest resolution used in the investigations was 0.01 mm, set for the antenna, and 1 mm, set for the male numerical model. The uncertainty of numerical simulations was estimated as ±15–25% (K = 1) and was within the range accepted by international standards [21,22,23]. 

## 3. Results 

### 3.1. EMF Emitted by a UHF RFID Gun

**Figure 4:**
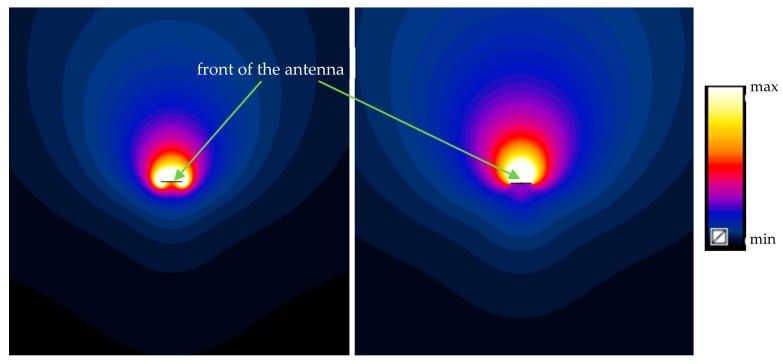
Magnetic (**left**, min–max: 0.0004–0.4 A/m) and electric (**right**, min–max: 0.01–100 V/m) field distribution in a plane perpendicular to the UHF RFID gun antenna plane at a radiated power of 1 W.

Figure 4 presents magnetic and electric field distributions in the vicinity of the modeled and investigated UHF RFID gun at the reader’s radiated power of 1 W as a cross-section distribution along the plane perpendicular to the antenna plane.

**Figure 5:**
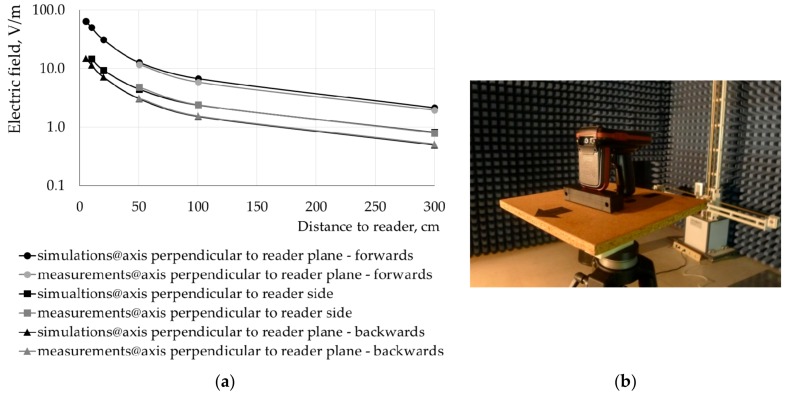
Electric field distribution near the UHF RFID gun reader normalized to a power of 1 W in the front or back of (along an axis perpendicular to the reader plane) and at the side of (perpendicular to the reader’s edge) the reader evaluated by measurements inside the semi-anechoic chamber and by numerical simulations (**a**) and the facilities used while the performing measurements inside the semi-anechoic chamber (**b**).

The numerical model of the UHF RFID gun reader was validated by comparing the electric field distribution near the reader obtained by measurements inside a semi-anechoic chamber and numerical simulations (Figure 5). The measurements to obtain the spatial distribution of EMF emitted by an RFID gun were performed in a semi-anechoic chamber (dimensions of 9.76 × 6.71 × 6.10 m) covered by a foam based radiofrequency absorber material (RANTEC Ferrosorb300), specified to have a reflection/absorption coefficient of −18 dB at the frequency of 865 MHz. The device under test was mounted on a manual positioning device, allowing the device to be rotated in two orthogonal planes and permitting the measuring antenna to sample the radiation pattern at any angle. A positioner with an EMCO 1050 and EMCO 1060-1.2 motor allowed changes of the measuring antenna between the horizontal and vertical positions. The measurements were carried out with an EMI Test Receiver ESIB26 (Rhode & Schwartz, Austria) with a frequency range of 20 Hz to 26.5 GHz, the measuring antenna was a Log-Per EMCO 3146 (linearly-polarised antenna) with a frequency range of 200 MHz to 1 GHz, and an RF generator R&S SMT02. The radiation pattern (maximum values from measurements for horizontal and vertical positions of the measuring antenna and the adjusted RFID antenna position) was sampled out around the antenna, starting in front of the UHF RFID antenna with angle step of 45° to cover major, lateral, and back radiation.

Electric field distribution was evaluated along the three axes passing through the center of the antenna of the UHF RFID gun: in the front and back of the reader plane (perpendicular to it and at the side of the reader) perpendicular to the reader’s edge at distances of 50, 100, and 300 cm to the reader center. The results were normalized to an output power equal to the case considered in the numerical simulations (1 W). The differences in electric field values measured and numerically simulated did not exceed 10%.

### 3.2. EMF Exposure Evaluation by SAR Values

The results of the numerical simulations of SAR values related to EMF exposure near the UHF RFID guns were evaluated at an emission level equal to 1 W of radiated power (6-min continuous exposure being the worst-case exposure scenario with respect to exposure duration) and normalized to general public SAR limits (as shown in Table 1). The EMF exposure conditions were evaluated in a realistic scenario with respect to the RFID gun position, where the reading gun is next to the operator’s body or near the body of a bystander or scanned person.

The comparison of normalized SAR inside the male numerical model (normalized whole-body averaged SAR—NWBSAR; normalised localized SAR10g—NSAR10g in head, torso, palm, or arm in the case of exposure scenario E-Side-5 cm, and legs) is presented in Table 2.

The highest NWBSAR value—0.57—was found in the RFID gun user at exposure scenario C, where the UHF RFID gun is held in the lowered palm, and the gun antenna is located nearly in front of the body at the center of its height (Table 2). The highest NWBSAR value—0.16—in the scanned person/bystander was found in the pregnant female model (exposure scenario F) as well as the highest NSAR10g value in the head.

In the considered exposure scenarios, the highest localized NSAR10g values approaching ICNIRP’s general public limits were found in the palm of RFID gun user (NSAR10g values 0.95, 0.94, and 1.0 in exposure scenarios A, B, and C, respectively; which may be considered to be equal when uncertainty in SAR evaluation is counted). The highest SAR values were found in the palm because of the very short distance between the reader antenna and the palm during the use of handheld RFID guns. Because of the longer distance between the RFID gun antenna and other body sections, the localized NSAR10g values in the head, torso, and legs were significantly lower.

In the exposure cases related to the exposure of a person approaching an activated UHF RFID gun, the highest localized NSAR10g was found in the torso (0.41 in exposure scenario D) and the arm (0.27 in exposure scenario E), at the closest proximity to the RFID gun antenna.

## 4. Discussion

Among the key properties of any RFID system is the reading range, which indicates how far away the tag may be located and still be effectively identified (caught) by the reader. In practice, this defines how fast the reading process can be performed as well as the level of EMF exposure near the reader, which is higher as the reading range increases.

Active tags have a local power source (such as a battery) and may operate typically up to hundred meters away from the RFID reader, while the reading range of passive tags has a typical scale from several centimeters up to several dozens of centimeters, and no more than several dozens of meters [4].

The radiated power of the particular gun is fixed (to ensure the required reading range) and independent of the distance at which the particular tags are being read (i.e., independent of the physical distance to the tag caught at the moment). As a shorter reading range is required in particular RFID applications, the level of EMF emitted by the gun may be lower (in RFID guns, the level of radiated power may be adjustable by the user) [24,25].

The reading range of the UHF RFID system depends on the power radiated by the reader and the sensitivity of the searched passive tag (the sensitivity of the tag is the function of the design of the tag chip and the tag antenna gain). This may be expressed as the strength of the electric field caused by the emission from the reader antenna, which needs to expose (energise) the tag to read (catch) it. The minimum electric field strength (*E*_tag_) needed to power up (read) the UHF RFID tag of a particular sensitivity (*P*_tag_), defined by its physical structure, can be expressed as follows [26]:

(1) $$E_{\mathrm{tag}}=\frac{4\pi }{\lambda }\sqrt{30P_{\mathrm{tag}}}$$

The data sheets of commercially available UHF RFID passive tags of various physical structures report their different sensitivity: *P*_tag_ ranges from 0.0001 W (−10 dBm) for older solutions, up to 0.00001 W (−20 dBm) for the latest ones. Using the formula expressed by Equation (1), the minimum strength of the electric field emitted from the RFID guns operating at a frequency of 865 MHz, needed to energize passive tags of various physical structures, was evaluated for the typical range of tag sensitivity 0.0001–0.00001 W (Table 3).

The distribution of the strength of the magnetic and electric fields along an axis perpendicular to the reader antenna plane, near the investigated UHF RFID gun reader (at least 10 cm away from the antenna) shown in Figure 2, can be estimated by the following formulas:

(2) $$H=\frac{2.2}{r}\sqrt{P}$$

(3) $$E=\frac{680}{r}\sqrt{P}$$

where *H* is the strength of the magnetic field in ampere per meter, *E* is the strength of electric field in volt per meter, *r* is the distance from the UHF RFID reader antenna in centimeters, *P* is the radiated power in watts, and 2.2 [A/W^0.5^] and 680 [V/W^0.5^] coefficients (power regression) were derived from the field distributions in the front of antenna (as shown at Figure 5).

Based on Formulas (1) and (3), the dependence of the minimum power of EMF emitted by the UHF RFID gun (*P*_min_) required to produce an electric field with the minimum strength required to read a particular tag (*E*_tag_) at a distance from the gun equal to the reader’s reading range (RR) can be expressed by the following equation:

(4) $$P_{\min}={\left(\frac{E_{\mathrm{tag}}}{680}RR\right)}^{2}$$

Thus, the minimum power (*P*_min_) needed to be radiated from the investigated UHF RFID gun for different reading ranges (RR), and tags sensitivity (*E*_tag_) were estimated, as shown in Table 4.

The obtained results show that doubling the reading range of the investigated UHF RFID reader requires the power radiating from the reader to be approximately four times higher. This also means the level of EMF exposure doubles near the reader, which doubles its reading range.

The minimum radiated power for the investigated UHF RFID gun related to the particular reading range depends heavily on the tag sensitivity (*P*_tag_), which defines the minimum electric field strength (*E*_tag_) needed to read a passive tag. When the use of a UHF RFID gun maintains the same reading range by delivering an electric field strength (*E*_tag_) sufficient to read used tags with differing sensitivities, then 0.8, 1.0, 1.3, 1.6, and 2.0 V/m needs 1.8-, 2.7-, 4.6-, 7-, and 11-times higher power from the reader, respectively, in comparison to the use of the most sensitive tags that require a minimum electric field strength of 0.6 V/m. Therefore, in order to reduce the level of exposure to EMF near a UHF RFID gun, the use of tags with high sensitivity (i.e., with low *E*_tag_) allows the level of power radiated from RFID reader to be reduced significantly in comparison with the case of using low sensitivity tags (i.e., with high *E*_tag_), while maintaining the required reading range.

Table 5 presents the level of emissions from the UHF RFID gun (radiated power in watts) that cause SAR values equal to ICNIRP’s general public limits in the considered exposure situations. It was found that in exposure scenarios A, B, and C, the localized NSAR10g in the palm of the user of a UHF RFID gun reader may exceed general public limits even at 1 W of radiated power, which is equal to just 50% of the radiated power allowed in Europe from non-registered RFID use. UHF RFID gun readers with a 1 W level of EMF emission are characterized by a reading range of 350–1100 cm, depending on the sensitivity of particular tags (Table 3).

The worst-case exposure scenario with respect to SAR evaluation may be counted as 6 min of continuous emission at maximum output power, though a more typical exposure situation is with a shorter exposure duration. In addition, when the required reading range is shorter, the level of radiated power may also be lowered at the stage of manufacturing or use (in some UHF RFID gun readers, the level of radiated power is adjustable by the user). 

Under real life conditions of using RFID guns, the SAR values received by the user may be slightly lower than presented in this study because the elements of antenna housing, its fixation, gun handle, and monitor were not included in the RFID gun numerical model, but they may decrease the level of EMF affecting the user. On the other hand, RFID guns with emission levels of multi-watts may be used in various locations without special permission for use (up to 2 W in Europe, established by ETSI/EN 302-208 V3.1.1:2016 [15], being a harmonized standard with Directive 2014/53/EU [14,15]; and 4 W in USA [27]), and even of higher levels of emission from devices used following special administrative rules.

In both cases, considering realistic exposure, at the very least, UHF RFID guns radiating over 1 W need to be considered as sources of potential EMF exposure to the user, causing SAR values in the palm that exceed ICNIRP’s general public limits (limits used in many countries as the EMF exposure evaluation criterion). The use of UHF RFID guns with a reading range of 5–15 m, which may need the use of radiated power at a level of 2–20 W (depending on the sensitivity of the searched tags) may cause the SAR values to exceed the general public limits not only in the palm of the user but also in the torso of the user, bystander, or a scanned person. At a level of radiated power exceeding 5 W, occupational exposure limits may also be exceeded in the palm, and at above 10 W of emission, also in the torso of the UHF RFID gun users.

On the other hand, RFID reader devices may also be equipped with other systems emitting radiofrequency EMF (e.g., Wi-Fi, GPS, Bluetooth, GSM, LTE, GPRS, HSDPA, WCDMA), which may increase SAR in users that may be activated simultaneously with the reader RFID antenna.

In the investigated exposure scenarios, the posture of the user of an RFID gun reader corresponds to the typical postures of users. It is not easily to reduce SAR values from RFID gun readers in palms. The first idea for how to lower the EMF exposure levels in the head and torso is to increase the distance between the EMF source and the user. The SAR values can be reduced by increasing that distance, but the weight of the gun (0.7 kg in the analyzed case) would be associated with increased fatigue in the limb holding the reader and reduce the work comfort.

Discussing the environmental influence of the use of UHF RFID guns, the safety of the vulnerable population also needs to be considered. The Occupational Health and Safety Framework Directive 89/391/EEC requires, in Article 15 about risk groups, that “Particularly sensitive risk groups must be protected against the dangers that specifically affect them.” According to European Directive 2013/35/EU on the protection of workers against EMF hazards in the work environment (Article 4.5), during the risk assessment process, the employer is obliged to investigate indirect effects from EMF exposure, such as interference from medical electronic equipment and devices (including cardiac pacemakers and other implanted devices) [14]. The possibility of interference from an electronic device depends on the EMF exposure level and the electromagnetic performance of the device, its settings, and the method of implantation. The clinical relevance of such an EMF exposure effect may depend on the duration of exposure, but also the health status of a particular user of medical implants. 

The evaluation of electromagnetic hazards for users of an active implantable medical device (AIMD) may be based on the approach that AIMDs manufactured for use in the European Union are expected to function as described in their product standards as long as the general public reference levels of Council Recommendation 1999/519/EC (Table 1) are not exceeded by the affecting EMF [28]. Given that at an emission level of 1 W, the electric field strength at distances up to 20 cm from the UHF RFID reader (18–55 times lower than its reading range) exceeds the level of these general public reference levels, the users of AIMD who are in close proximity to UHF RFID guns should be treated as individuals at particular risk. The other references for an evaluation of situations in which AIMD users may be at particular risk may be taken from the rules of the AIMD immunity tests regarding disturbances in their work from electromagnetic exposure (following the protocol provided by EN 60601-1-2:2015 [29], the tests are required at exposure levels of 3 and 10 V/m levels, at the 80 MHz to 2.7 GHz frequency band). As RFID tags of low sensitivity need to be energized by an electric field with a minimum strength approaching 2 V/m, the EMF exposure from an RFID reader that is not compliant with the lower level of the mentioned immunity tests may be found even at a distance from the gun of approximately 50% of the reading range.

Employees who are AIMD users need to be aware of the particular risks that they might encounter during their work and should be instructed on how to deal with equipment that is a source of significant EMF exposure in a safe manner. Generally, this means that employees who are AIMD users should be informed about the interference distances or zones of such equipment. Devices should be marked with symbols indicating a threat to the proper functioning of AIMD (e.g., according to ISO 7010:2011 [30]).

## 5. Conclusions

The EMF exposure conditions were evaluated in a worst-case scenario with respect to the EMF exposure duration, and in realistic scenarios with respect to the RFID gun position, where the reading gun is next to the operator’s, a bystander’s, or the scanned person’s body.

The results of this study show that the EMF exposure while using UHF RFID gun readers, evaluated by numerical simulations of realistic exposure scenarios, do not cause SAR values exceeding general public limits (set by ICNIRP guidelines and non-binding European Council Recommendation 1999/519/EC) when the emitted power does not exceed 1 W (which corresponds to a reading range of 3.5–11 m, depending on the sensitivity of the searched tags).

The use of UHF RFID guns with a reading range of 5–15 m, which may need the use of emitted power at the level of 2–20 W (depending on the sensitivity of the searched tags) may cause SAR values exceeding general public limits, not only in the palm of the user but also in the torso of the user, a bystander or a scanned person. At a level of over 5 W of radiated power, occupational exposure limits may also be exceeded in the palm, and at over 10 W, also in the torso of the UHF RFID gun users or bystanders.

Given that electric field strength at distances of up to 20 cm from a UHF RFID reader with a radiated power of 1 W exceeds the level of general public reference levels set by Council Recommendation 1999/519/EC, any users of AIMD and pregnant women who are in close proximity to a UHF RFID gun reader should be treated as individuals at particular risk.

Only UHF RFID guns emitting below 1 W may be considered as environmentally insignificant EMF sources.

## Figures and Tables

**Table 1:** International Commission on Non-ionizing Radiation Protection (ICNIRP)-based limitation of exposure to electromagnetic field (EMF) at frequencies used by ultra-high frequency radiofrequency identification (UHF RFID) systems (900 MHz).

Exposure Scenario	Basic Restrictions @ UHF RFID Frequencies			Reference Levels @ UHF RFID Frequencies	
	Whole-Body Average WBSAR	Local Head/Torso SAR10g	Local limb SAR10g	Electric Field Strength	Magnetic Field Strength
	W/kg	W/kg	W/kg	V/m	A/m
Occupational	0.4	10	20	90	0.2
General public	0.08	2	4	40	0.1

Note: 1. All WBSAR and SAR10g values are to be averaged over a 6-min period. 2. WBSAR values are to be averaged over the entire body. 3. SAR10g values are to be averaged over a 10 g mass of any continuous tissue.

**Table 2:** Comparison of the whole body averaged and localized SAR under exposure to an EMF emitted by a modeled UHF RFID reader, at 1 W of radiated power and a frequency of 865 MHz (6-min continuous exposure).

Exposure scenario	NWBSAR	NSAR10g				
		Head	Torso	Palm	Arm	Legs
A–Head-20cm	0.40	0.041	0.017	0.95	-	0.00060
B–Chest-20cm	0.45	0.027	0.17	0.94	-	0.0020
C–Hip-16cm	0.57	0.010	0.18	1.0	-	0.0063
D–Chest-5cm	0.14	0.056	0.41	-	0.013	0.0043
E–Side-5cm	0.090	0.025	0.22	-	0.27	0.0026
F-Chest-5cm	0.16	0.15	0.32	-	0.016	0.0012
G-Side-5cm	0.11	0.070	0.14	-	0.26	0.0044

Note: NSAR values were normalized to SAR limits used in the evaluation of general public EMF exposure; SAR limits used in the evaluation of occupational EMF exposure were 5-times higher; NWBSAR—SAR averaged over the entire body; NSAR10g—localized SAR averaged over any 10 g of tissue in the head, torso, palm, or legs; A–C—exposure scenarios in which SAR values evaluated in an RFID gun user (male model); D and E—exposure scenarios in which SAR values were evaluated in the scanned person or bystander (male model); F and G—exposure scenarios in which SAR values were evaluated in the scanned person or bystander (pregnant female model); underlined values—maximum values of localized SAR in particular exposure scenarios; values in bold—maximum of NWBSAR and NSAR10g values in all the analyzed exposure scenarios

**Table 3:** Minimum strength of the electric field (*E*_tag_) required to read the UHF RFID tags of differing sensitivities (*P*_tag_), operating at a frequency of 865 MHz.

Tag Sensitivity (*P*_tag_), W (dBm)	Minimum Strength of the Electric Field (*E*_tag_) Required to Read the Tag, V/m
0.00001 (−20)	0.6
0.000016 (−18)	0.8
0.000025 (−16)	1.0
0.00004 (−14)	1.3
0.000063 (−12)	1.6
0.0001 (−10)	2.0

**Table 4:** Relation between the minimum radiated power (*P*_min_) and reading range (RR) of the investigated UHF RFID reader used with tags of different sensitivity.

Reading Range (*RR*), cm	Minimum Power Radiated from the UHF RFID Reader to Energize Particular Tags (*P*_min_), W					
	The Sensitivity of Used Tags, Expressed by the Minimum Strength of the Electric Field Required to Energize Them (*E*_tag_), V/m					
	0.6	0.8	1.0	1.3	1.6	2.0
50	0.002	0.004	0.005	0.009	0.014	0.022
100	0.008	0.014	0.022	0.037	0.055	0.087
150	0.018	0.031	0.049	0.082	0.13	0.20
200	0.031	0.055	0.087	0.15	0.22	0.35
300	0.070	0.12	0.20	0.33	0.50	0.78
400	0.13	0.22	0.35	0.59	0.89	1.2
500	0.20	0.35	0.54	0.91	1.4	2.2
600	0.28	0.50	0.78	1.3	2.0	3.1
700	0.38	0.68	1.1	1.8	2.7	4.2
800	0.50	0.89	1.4	2.3	3.5	5.5
1000	0.78	1.4	2.2	3.7	5.5	8.7
1500	1.8	3.1	4.9	8.2	12	19

Note: analyzed UHF RFID GUN antenna—microstrip patch antenna with a patch of 99.6 × 122.5 mm placed on substrate and a ground of 131.5 × 138.3 mm, operated at a frequency of 865 MHz; values in bold—the level of emission which needs in Europe administrative permission for use in RFID systems.

**Table 5:** The level of EMF emission, at 856 MHz from a UHF RFID reader, which causes SAR values in users or bystanders equal to general public limits in the considered exposure scenarios (6 min of continuous exposure).

Exposure Scenario	Radiated Power, W					
	WB NSAR	NSAR10g				
		Head	Torso	Palm	Arm	Legs
A–Head-20cm	2.5	24	58	1.1	-	1700
B–Chest-20cm	2.3	38	6.0	1.1	-	570
C–Hip-16cm	1.8	97	5.5	0.98	-	160
D–Chest-5cm	6.9	18	2.4	-	78	230
E–Side-5cm	11	40	4.6	-	3.7	390
F-Chest-5cm	6.4	6.7	3.1	-	63	820
G-Side-5cm	9.1	14	7.3	-	3.8	230

Note: underlined values—minimum values of whole-body and localized SAR in all analyzed exposure scenarios; values in bold—the level of radiated power required to provide a reading range of 5–15 m.
